# Lifespan Extension and Sustained Expression of Stem Cell Phenotype of Human Breast Epithelial Stem Cells in a Medium with Antioxidants

**DOI:** 10.1155/2016/4591310

**Published:** 2016-10-11

**Authors:** Kai-Hung Wang, An-Pei Kao, Chia-Cheng Chang, Ta-Chin Lin, Tsung-Cheng Kuo

**Affiliations:** ^1^Department of Obstetrics and Gynecology, Kuo General Hospital, Tainan, Taiwan; ^2^Center for Reproductive Medicine, Kuo General Hospital, Tainan, Taiwan; ^3^Department of Laboratory Medicine, Kuo General Hospital, Tainan, Taiwan; ^4^Department of Research and Development, NeoAsia, Taipei, Taiwan; ^5^Department of Pediatrics and Human Development, Michigan State University, East Lansing, Michigan 48824, USA

## Abstract

We have previously reported the isolation and culture of a human breast epithelial cell type with stem cell characteristics (Type I HBEC) from reduction mammoplasty using the MSU-1 medium. Subsequently, we have developed several different normal human adult stem cell types from different tissues using the K-NAC medium. In this study, we determined whether this low calcium K-NAC medium with antioxidants (N-acetyl-L-cysteine and L-ascorbic acid-2-phosphate) is a better medium to grow human breast epithelial cells. The results clearly show that the K-NAC medium is a superior medium for prolonged growth (cumulative population doubling levels ranged from 30 to 40) of normal breast epithelial cells that expressed stem cell phenotypes. The characteristics of these mammary stem cells include deficiency in gap junctional intercellular communication, expression of Oct-4, and the ability to differentiate into basal epithelial cells and to form organoid showing mammary ductal and terminal end bud-like structures. Thus, this new method of growing Type I HBECs will be very useful in future studies of mammary development, breast carcinogenesis, chemoprevention, and cancer therapy.

## 1. Introduction

Stem cells are undifferentiated cells with high self-renewal and differentiation ability. Stem cell research has emerged as a major focus in biomedical research for its potential in cell-based reparative and regenerative medicine and for its key role in carcinogenesis.

In the breast, there are two epithelial cell lineages, myoepithelial and luminal epithelial cells, which are derived from stem cells [1, 2]. These cells constitute the mammary gland, forming the ductal and lobuloalveolar structures [1, 3, 4]. In women, the mammary gland is a dynamic organ that undergoes a series of changes from pregnancy, lactation, and involution [5, 6]. Stem cells are also believed to be the origin of breast cancer [7]. Therefore, breast stem cells are important for studies of the mechanism of mammary development, carcinogenesis chemoprevention, and cancer therapy.

We have previously developed a cell culture method for isolation and culture of 2 types of normal human breast epithelial cells (HBECs) from reduction mammoplasty [8]. These two types of cells, Type I and Type II HBECs, have been extensively characterized and found to differ substantially in phenotypes. Type II HBECs express maspin and basal epithelial cell marker, cytokeratin 14 (CK14) [8]. In contrast, Type I HBECs express estrogen receptors and luminal epithelial cell markers, that is, epithelial membrane antigen (EMA), CK18, and CK19 [8]. Significantly, Type I HBECs display many stem cell characteristics. These include (1) the deficiency in gap junctional intercellular communication (GJIC) [7, 8]; (2) the expression of the embryonic and adult stem cell marker, Oct-4; (3) the ability to differentiate into basal (Type II HBECs) and luminal (acini-forming) epithelial cells [8]; (4) the ability of anchorage independent growth and to form budding/ductal organoids [9]. Furthermore, Type I HBECs were found to be more susceptible to telomerase activation, immortalization, and neoplastic transformation, a strong evidence for the stem cell theory of carcinogenesis (see references for these characterizations in [7]). The step-wise neoplastic transformation of stem cells provides an in vitro model of breast cancer progression [9], including the emergence of breast cancer stem cell marker CD44^+^/CD24^−^ [10, 11].

Although these HBECs are useful for studies of mammary biology and carcinogenesis, these cells cultured in the MSU-1 medium had limited proliferation potential (~3 passages) [8]. Subsequent to these studies of HBECs, we have reported the development of several human adult stem cells from various tissues, that is, liver, gastric, amniotic fluid, and endometrial and adipose-derived mesenchymal stem cells [12–16]. The success in developing these stem cells is mainly ascribed to the use of a low calcium medium (0.09 mM) (the K-NAC medium) containing antioxidants, N-acetyl-L-cysteine (NAC), and L-ascorbic acid-2-phosphate (Asc-2P), which change the cellular redox state and facilitate the expression of major stem cell transcription factors. In this study we carried out experiments to determine if the K-NAC medium is a better medium to increase the self-renewal ability of Type I HBECs while preserving the expression of stem cell characteristics of these cells.

## 2. Materials and Methods

### 2.1. Culture of Human Breast Epithelial Cells

Three normal human breast epithelial cell (HBEC) cultures (designated HBEC30, HBEC31, and HBEC35) were isolated from three different women (aged 23, 21, and 43, resp.) during reduction mammoplasty at Sparrow Hospital in Lansing, MI. Patients' written consent was received and the use of HBEC was approved by the institutional review board of Michigan State University. The MSU-1 medium with supplements and the procedure used to develop the two types (Type I, Type II) of normal HBECs from the initial cultures have been reported previously [8]. After one week, Type I HBECs were trypsinized for experiments or storage in liquid nitrogen. Three cell culture media were used in this study, the MSU-1 medium [8] with or without supplementation of 0.2 mM Asc-2P (Sigma-Aldrich, St. Louis, MO, USA) and 2 mM NAC (Sigma A8199) (without specification, MSU-1 medium refers to the latter without supplementation of NAC and Asc-2P), and a modified MCDB 153 medium (Keratinocyte-SFM, GIBCO-Invitrogen Corporation, Carlsbad, CA) supplemented with 0.2 mM Asc-2P and 2 mM NAC (referred to as K-NAC medium) [16]. The growth factors/hormones for the K-NAC medium are rEGF (5 ng/mL), bovine pituitary extract (50 *μ*g/mL), insulin (5 *μ*g/mL), hydrocortisone (74 ng/mL), and 3,3′,5-triiodo-D.L.-thyronine (T_3_) (6.7 ng/mL).

### 2.2. Cumulative Population Doubling Level

Cumulative population doubling level (cpdl) in continual subculture and growth from a known number of cells (1 × 10^5^) was calculated to determine the proliferation potential of Type I HBECs. The cpdl in each subcultivation was calculated from the cell count by using the equation: ln⁡(*N*_*f*_/*N*_*i*_)/ln⁡2, where *N*_*i*_ and *N*_*f*_ are initial and final cell numbers, respectively, and ln is the natural log.

### 2.3. Gap Junctional Intercellular Communication

The gap junctional intercellular communication (GJIC) was studied by the scrape loading/dye transfer technique developed in our laboratory [17]. The dye transfer between cells was observed using a Nikon Eclipse TE300 UV fluorescence microscope and recorded by a digital camera connected to a computer.

### 2.4. Immunochemical Analysis

For immunostaining, the cells grown in 35 mm plates were washed with phosphate buffered saline (PBS) and fixed by 4% paraformaldehyde in PBS for 20 min. After rinsing with PBS, the cells were permeabilized (0.5% triton x-100, 2% BSA, and 0.05% NaN3 in PBS) for 10 minutes. The cells were then incubated with the primary antibody [anti-Oct-4 (1 : 200 dilution, Chemicon Company, Temecula, CA), anti-CK18 (1 : 200, Sigma-Aldrich, St. Louis, MO, USA), anti-CK19 (1 : 200, Sigma-Aldrich, St. Louis, MO, USA), anti-CK-14 (1 : 200, Sigma-Aldrich, St. Louis, MO, USA), anti-Maspin (1 : 100, BD Biosciences-Pharmingen, San Jose, CA USA), and anti-EMA (1 : 100) which was a gift from Dr. M. G. Ormerod (Institute of Cancer Research, Royal Cancer Hospital, Sutton, Surrey, UK)] in PBS/triton/BSA at 25°C overnight. The following day, the cells were incubated with a secondary antibody conjugated with FITC in PBS/triton/BSA buffer for 1 hr at 25°C. After washing with PBS thoroughly, the phase image and fluorescence of cells were observed and recorded using a Nikon Eclipse TE 300 microscope connected to a digital camera and computer.

### 2.5. Western Blotting

The proteins were extracted with 20% SDS lysis solution containing several protease and phosphatase inhibitors (1 mM phenylmethylsulfonyl fluoride, 1 mM leupeptin, 1 mM antipain, 0.1 mM aprotinin, 0.1 mM sodium orthovanadate, and 5 mM sodium fluoride). Protein concentrations were measured using Biorad Protein Quantification kit (Biorad, CA, USA). Same amounts of protein (15 *μ*g/lane) were separated by 12% SDS-PAGE and transferred from the gel to PVDF membranes (Millipore Corp, Bedford, MA). Immunoblotting was carried out using monoclonal antibody (anti-Oct-4, anti-CK18, anti-CK19, anti-CK-14, anti-Maspin, and anti-EMA). This was then followed by incubation with horseradish peroxidase-conjugated secondary antibody and detected with the ECL chemiluminescent detection reagent (Amersham Co., IL, USA). The membranes were exposed to X-ray film for 15 s to 1 min.

### 2.6. Induction of Differentiation of Type I HBEC into Type II HBEC

Type I HBECs were inoculated in the K-NAC medium in the presence of cholera toxin (1 ng/mL, Sigma-Aldrich, St. Louis, MO, USA) to induce the differentiation of Type I HBECs to Type II HBECs for 14 days. The medium was changed every other day.

### 2.7. Mammary Ductal Organoid Formation by Coculture of Two HBEC Cell Types

Type I and Type II HBECs in 1 : 2 cell number ratio were inoculated in poly-D-lysine (Sigma-Aldrich, St. Louis, MO, USA) coated plate. The mixture of cells started to form organoids overnight and were allowed to grow for 4 weeks.

### 2.8. Statistical Analysis

Results shown were obtained from at least three separate experiments. The significance of difference between treatments was assessed by the Mann-Whitney test of nonparametric statistics and was carried out using SPSS for Windows 13.0 statistics program. The *P* value <0.05 was considered to be significant. All statistical data are presented as mean ± SD.

## 3. Results

### 3.1. Morphology of Type I HBECs Cultured in Different Media

**Figure 1:**
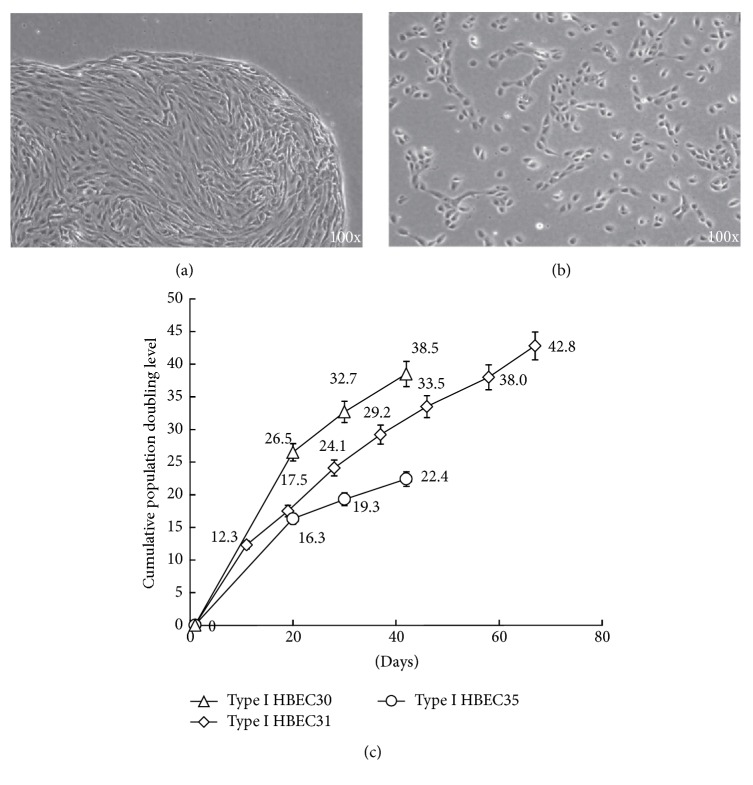
Type I HBECs plated and cultured in MSU-1 medium (a) formed colonies with smooth and restricted boundaries, the cells in colonies were contiguous and variable-shaped, and Type I HBECs grown in K-NAC medium (b) were dispersed. (c) Proliferation potential of Type I HBECs cultured in K-NAC medium: cpdl = 38.5, 42.8, and 22.4, respectively, for HBEC30, HBEC31, and HBEC35.

Normal human breast epithelial cells were developed from reduction mammoplasty tissues of three different women. Type I HBECs initially developed in the MSU-1 medium with 5% FBS were subcultured and grown in two different media, that is, MSU-1 and K-NAC medium. Morphologically, Type I HBECs in MSU-1 medium formed colonies with contiguous variable-shaped cells and smooth restricted boundaries in one week (Figure 1(a)). In contrast, Type I HBECs grown in the K-NAC medium were dispersed and more uniform in cell shape (cobble stone-like) (Figure 1(b)). The morphologies were reversible upon change of different medium (data not shown).

### 3.2. Proliferation Potential for Type I HBECs Cultured in K-NAC Medium

Type I HBECs cultured in the K-NAC medium showed higher proliferation ability and grew for more than 40 days in culture, achieving a total of 38.5 cpdl in 42 days, 42.8 cpdl in 67 days, and 22.4 cpdl in 42 days, respectively, for HBEC30, HBEC31, and HBEC35 (Figure 1(c)). The proliferation ability of these cells in the K-NAC medium is much higher than that for Type I HBECs grown in the MSU-1 medium we observed before.

### 3.3. Antioxidants Enhanced Proliferation of Type I and Type II HBECs

**Figure 2:**
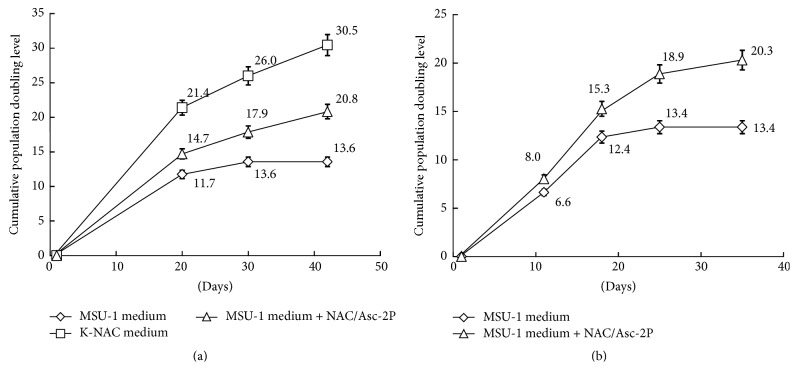
Antioxidants enhanced proliferation of Type I HBECs. (a) The average cpdl for HBEC30, HBEC31, and HBEC35 developed in the K-NAC medium (cpdl 30.5) was higher than that in MSU-1 medium and the MSU-1 medium with NAC/Asc-2P (cpdl 13.6 and 20.8, resp.) (*P* < 0.05). (b) The average cpdl of Type II HBECs developed in the MSU-1 medium with NAC/Asc-2P (cpdl 20.3) was higher than that in MSU-1 medium (cpdl 13.4) (*P* < 0.05).

Type I HBECs were cultured in three kinds of medium, that is, MSU-1, MSU-1 with NAC/Asc-2P, and K-NAC medium. After 3 passages, the average cpdl of Type I HBECs (HBEC30, HBEC31, and HBEC35) developed in the K-NAC medium (cpdl 30.5) was higher than that in the MSU-1 medium and in the MSU-1 medium with NAC/Asc-2P (cpdl 13.6 and 20.8, resp.) (Figure 2(a)). The average cpdl of Type II HBECs developed in the MSU-1 medium with NAC/Asc-2P (cpdl 20.3) was higher than that in the MSU-1 medium (cpdl 13.4) (Figure 2(b)). These results indicate that NAC and Asc-2P could significantly enhance the proliferation potential of Type I and Type II HBECs.

### 3.4. Gap Junctional Intercellular Communication of Type I HBECs Cultured in K-NAC Medium

**Figure 3:**
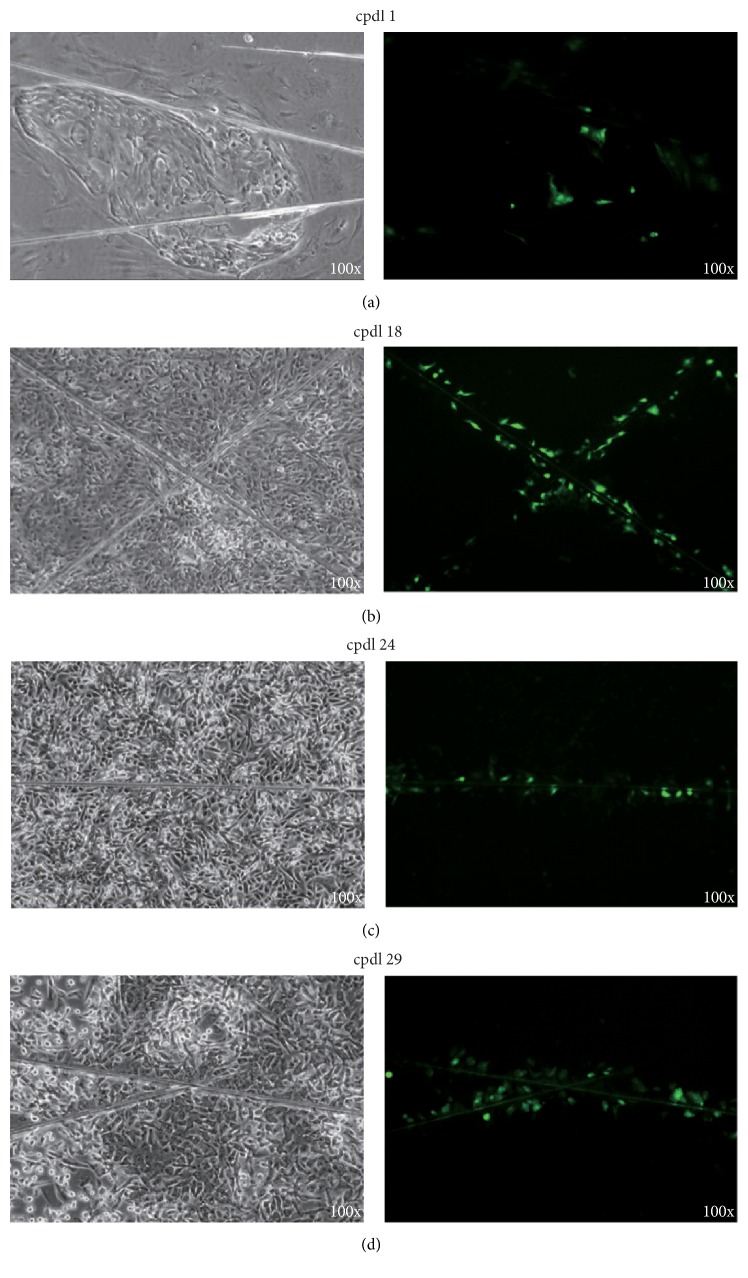
Gap junctional intercellular communication of Type I HBECs cultured in K-NAC medium studied by the Lucifer Yellow scrape loading/dye transfer technique. The results show that Type I HBECs in early and middle passage (cpdl = 1, 18, and 24) (a–c) were deficient in dye transfer. In later passage (cpdl 29) (d), the Type I HBECs showed very limited dye transfer. The same area of cells was observed under a phase contrast (left) or fluorescence (right) microscope.

Cancer cells and many human adult stem cells have been shown to be deficient in gap junctional intercellular communication (GJIC) [7, 18]. On the contrary, the normal somatic cells are competent in GJIC. To determine if Type I HBECs developed in the K-NAC medium are capable of GJIC, the scrape loading/dye transfer technique [17] was used to examine confluent Type I HBECs grown in the K-NAC medium at different cpdl. The results revealed that Type I HBECs developed in the K-NAC medium at different cpdl (1, 18, 24, and 29) were deficient in GJIC (Figures 3(a)–3(d)), similar to those Type I HBECs developed in the MSU-1 medium.

### 3.5. Expression of Stem Cell Markers of Type I HBECs Cultured in K-NAC Medium

**Figure 4:**
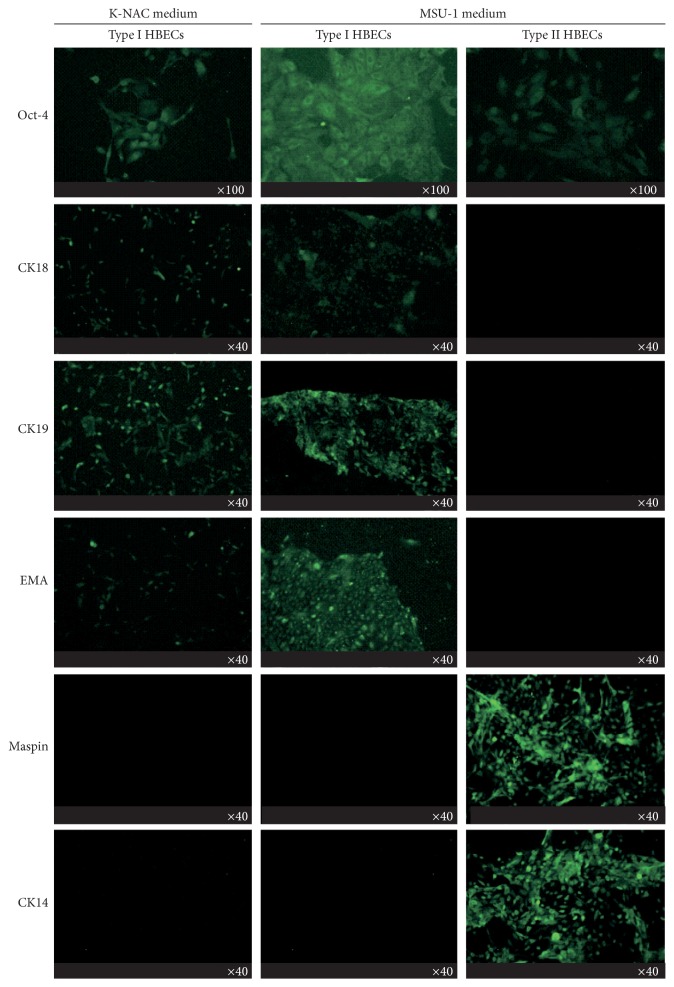
By immunostaining, Type I HBECs cultured in K-NAC and MSU-1 medium were found to express Oct-4, CK18, CK19, and EMA but not maspin and CK14. Type II HBECs expressed Oct-4, maspin, and CK14 but not CK18, CK19, and EMA as detected by immunostaining.

**Figure 5:**
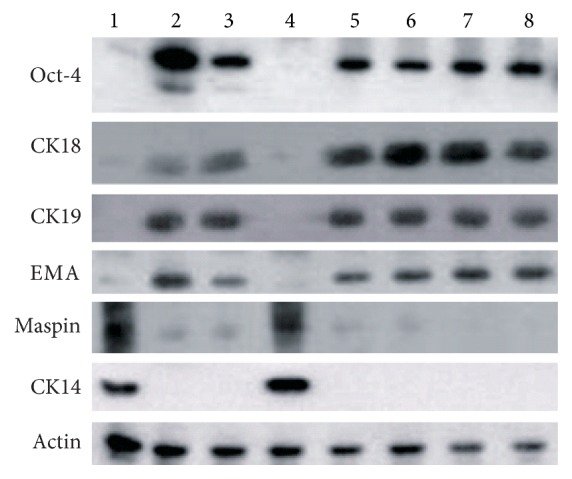
Western blot analysis of Oct-4, CK18, CK19, EMA, maspin, and CK14 expression in HBECs. Lane 1: Type II HBEC30 developed in the MSU-1 medium; Lane 2: Type I HBEC30 developed in the MSU-1 medium; Lane 3: Type I HBEC30 developed in the K-NAC medium (cpdl = 27); Lane 4: Type II HBEC31 developed in the MSU-1 medium; Lane 5: Type I HBEC31 developed in the MSU-1 medium; Lane 6: Type I HBEC31 developed in the K-NAC medium (cpdl = 18); Lane 7: Type I HBEC31 developed in the K-NAC medium (cpdl = 24); Lane 8: Type I HBEC31 developed in the K-NAC medium (cpdl = 29).

By immunostaining, the Oct-4 was found to be expressed in Type I HBECs developed in the K-NAC medium. CK18, CK19, and EMA which are specifically expressed in luminal epithelial cells were also expressed in these Type I HBECs. These biomarkers were also expressed in Type I HBECs developed in the MSU-1 medium as we reported before [8]. Type I HBECs cultured in the K-NAC and MSU-1 media did not express maspin and CK14 (basal epithelial cell markers), in contrast to Type II HBECs which were strongly positive in expression (Figure 4). Maspin, a gene for protease inhibitor, has been considered as tumor suppressor gene based on differential display studies showing its expression in normal cells, but not in tumor and stem cells [19–21]. To confirm these results from immunostaining studies, two cell lines (HBEC30 and HBEC31) developed in 2 different media were tested for the expression of 6 markers (Oct-4, CK14, CK18, CK19, EMA, and maspin) by western blot analysis. These results confirm that Type I HBECs grown in the K-NAC and MSU-1 medium expressed all markers except CK14 and maspin protein (Figure 5). Oct-4, the universal stem cell marker, consistently expressed Type I HBECs developed in the K-NAC medium (Figure 5). These data clearly indicate that Type I HBECs developed in the K-NAC medium expressed Oct-4, CK18, CK19, and EMA but not CK14 and maspin, similar to Type I HBECs developed in the MSU-1 medium.

### 3.6. Type I HBECs Differentiated into Type II HBECs by Cholera Toxin

**Figure 6:**
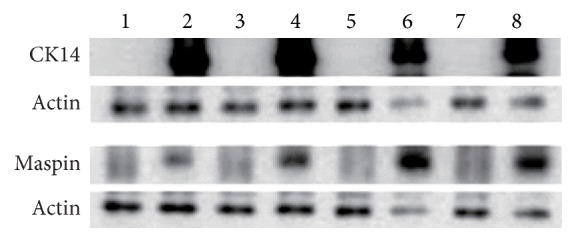
Differentiation induction of Type I HBECs developed in the K-NAC medium by cholera toxin into Type II HBECs. Lane 1: Type I HBEC30 developed in the MSU-1 medium; Lane 2: Type II HBEC30 developed in the MSU-1 medium; Lane 3: Type I HBEC30 developed in the K-NAC medium; Lane 4: Type I HBEC30 developed in the K-NAC medium with cholera toxin (14 days); Lane 5: Type I HBEC31 developed in the MSU-1 medium; Lane 6: Type II HBEC31 developed in the MSU-1 medium; Lane 7: Type I HBEC31 developed in the K-NAC medium; Lane 8: Type I HBEC31 developed in the K-NAC medium with cholera toxin (14 days).

The early passage Type I HBECs (HBEC30 and HBEC31) were cultured in the K-NAC medium in the absence or presence of cholera toxin. After 14 days' culture in medium with cholera toxin, the typical morphology of Type I HBECs had been changed into that of Type II HBEC.  Figure 6 showed that Type I HBECs developed in the K-NAC medium with cholera toxin expressed CK14 and maspin (Figure 6, Lanes 4 and 8), similar to Type II HBECs (Figure 6, Lanes 2 and 6). The results provide evidence that cholera toxin is capable of inducing differentiation of Type I HBECs into Type II HBECs as reported before [8].

### 3.7. In Vitro Formation of Mammary Organoids

**Figure 7:**
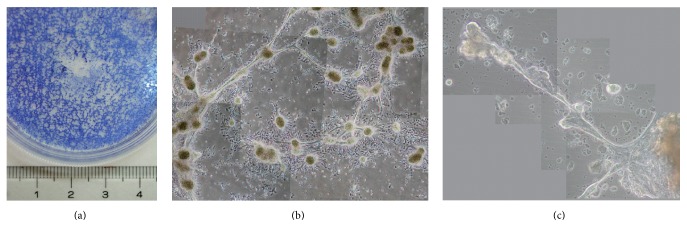
Two types of HBECs (Type I HBECs developed in the K-NAC medium and Type II HBECs in 1 : 2 ratio) were inoculated in a poly-D-lysine coated plate. (a, b) After plating for 14 days, these cells formed mammary organoids. (c) After incubation for 28 days, these organoids were found to develop many mammary ductal and terminal end bud-like structures.

Type I HBECs developed in the K-NAC medium and Type II HBECs were trypsinized and cocultured in 1 to 2 ratio on poly-D-lysine coated plates. These cells start to aggregate overnight and formed mammary organoids in 2 weeks (Figures 7(a) and 7(b)). The ability of the two types of HBECs to form mammary organoids on the poly-D-lysine coated plates was similar to that observed previously [9]. After incubation for 4 weeks, these mammary organoids were found to develop many mammary ductal and terminal end bud-like structures (Figure 7(c)).

## 4. Discussion and Conclusion

The major reason that prompted this study was our dissatisfaction with the low proliferation potential of Type I HBECs in the MSU-1 medium and our findings that the K-NAC medium was able to support the growth of many human and mammalian adult stem cells with extended lifespan (e.g., human liver stem cells, 50 cpdl; human amniotic fluid-derived mesenchymal stem cells, about 78 cpdl; human adipose-derived mesenchymal stem cells, 35 cpdl) [12, 14, 16].

The comparative studies of the growth of Type I HBECs using these 2 media, MSU-1 and K-NAC, revealed some similarities and differences in phenotypes of these cells. The similarities include (1) deficiency in gap-junctional intercellular communication (Figure 3), a hallmark of many stem cells and tumor cells [7, 8, 15, 16, 18, 22, 23]; (2) the expression of stem cell and luminal epithelial cell markers (i.e., Oct-4, CK18, CK19, and EMA) (Figures 4 and 5) similar to previous studies [7]; (3) the induction of differentiation of Type I HBECs into Type II HBECs by cholera toxin, a cyclic AMP-inducing agent (Figure 6); (4) the ability to form mammary organoids showing ductal and terminal end bud-like structures on poly-D-lysine coated plates (Figure 7); and (5) the absence of the expression of CK14 and maspin. CK14 was specifically expressed in basal epithelial cells and Type II HBECs whereas maspin has been considered as a tumor suppressor gene.

The difference in phenotypic expression of Type I HBECs grown in MSU-1 and K-NAC medium has been observed. First, as shown in Figure 1, the morphology of cells and colonies was different. Unlike the contiguous variable-shaped cells and restricted colonies in MSU-1 medium, the cells grown in the K-NAC medium were dispersed and similar to Type II HBECs developed in the MSU-1 medium which were more uniform and cobble stone-shaped [8]. Second and the most significant difference is the proliferation potential. Type I HBECs in K-NAC medium consistently showed higher proliferation potential and shorter generation time (i.e., Type I HBEC30: 38.5 cpdl in 42 days after 3 passages, Type I HBEC31: 42.8 cpdl in 67 days after 7 passages, and Type I HBEC35: 22.4 cpdl in 42 days after 3 passages) (Figure 1(c)), compared to the same cells grown in the MSU-1 medium (13.6 cpdl in 40 days) (Figure 2(a)). The proliferation enhancing effect of K-NAC medium can be ascribed to the antioxidant Asc-2P and NAC which could change cellular redox and promote self-renewal of precursor cells [24]. In this study these supplements were also found to enhance the cpdl of Type I and Type II HBECs in the MSU-1 medium (Figures 2(a) and 2(b)). NAC is readily deacetylated in cells to yield L-cysteine, thereby enhancing the production of glutathione [25]. Asc-2P is a stable precursor to provide ascorbic acid in cell culture [26, 27]. Ascorbic acid can be antioxidative or prooxidative (i.e., reversible interconversion between ascorbate and dehydroascorbate) depending on its concentration and concentration of metal ions. The calcium concentration of K-NAC medium (0.09 mM) is lower than in MSU-1 medium (0.98 mM). This may also be responsible for the difference in proliferation potential, since high concentration of calcium may induce cell differentiation [28].

The major result of this study is the demonstration that K-NAC medium is a better medium to support the growth of Type I HBECs with high self-renewal ability and sustained expression of stem cell characteristics compared to our MSU-1 medium or those reported in literature. Therefore, the new method of growing Type I HBECs will be very useful in future studies of mammary development, breast carcinogenesis, chemoprevention, and cancer therapy.
